# Estimating mutation rate and characterising single nucleotide de novo mutations in pigs

**DOI:** 10.1186/s12711-025-00967-1

**Published:** 2025-04-14

**Authors:** Christina M. Rochus, Marije J. Steensma, Marco C. A. M. Bink, Abe E. Huisman, Barbara Harlizius, Martijn F. L. Derks, Richard P. M. A. Crooijmans, Bart J. Ducro, Piter Bijma, Martien A. M. Groenen, Han A. Mulder

**Affiliations:** 1 Wageningen University & Research Animal Breeding and Genomics, P.O. Box 338, 6700 AH Wageningen, The Netherlands; 2 Hendrix Genetics, P.O. Box 114, 5830 AC Boxmeer, The Netherlands; 3 Topigs Norsvin Research Center, Meerendonkweg 25, 5216 TZ Den Bosch, The Netherlands; 4 Present Address: The University of Edinburgh, The Roslin Institute Easter Bush Campus, Midlothian, EH25 9RG Scotland

## Abstract

**Background:**

Direct estimates of mutation rates in humans have changed our understanding of evolutionary timing and de novo mutations (DNM) have been associated with several developmental disorders in humans. Livestock species, including pigs, can contribute to the study of DNM because of their ideal population structure and routine phenotype collection. In principle, there is the potential for livestock populations to quickly accumulate new genetic variants because of short generation intervals and high selection intensity. However, the impact of DNM on the fitness of individuals is not known and with current genomic selection programs they cannot contribute to estimated breeding values. The aims of our project were to detect and validate single nucleotide DNM in two commercial pig breeding lines, estimate the single nucleotide mutation rate, and characterise DNM.

**Results:**

We sequenced (150 bp paired end reads, 30X coverage) 46 pig trios from two commercial lines. Single nucleotide DNM were detected using a trio-aware method. We defined candidate DNM as single nucleotide variants (SNVs) found in heterozygous state in trio-offspring with both trio-parents homozygous for the reference allele. In this study, we estimate a lower threshold of the DNM rate in pigs of 6.3 × 10^–9^ per site per gamete. Our findings are consistent with those from other mammals and those published for a small number of livestock species. Most DNM we detected were in introns (47%) and intergenic regions (49%). The mutational spectrum in pigs differs from that in humans and we found several DNM predicted to have an effect on animal’s fitness based on the base pair change and their location in the genome.

**Conclusions:**

With this study, we have generated fundamental knowledge on mutation rate in a non-primate species and identified DNM that could have an impact on the fitness of individuals.

**Supplementary Information:**

The online version contains supplementary material available at 10.1186/s12711-025-00967-1.

## Background

Spontaneous mutation of germline DNA is the source of new genetic variants and is continuously contributing to genetic variation [1]. While these new genetic variants, also known as de novo mutations (DNM), can contribute to a population’s ability to adapt, they can also disrupt the function of genes, affecting fitness of an individual. DNA sequencing of trios has allowed direct estimates of mutation rate and detection of DNM and has been facilitated progressively by the decreasing cost of sequencing. This in turn has allowed the study of the effects of new mutations and calibration of molecular clocks [2]. Detection of DNM in humans have highlighted the trade-off between adaptation and fitness. While accumulation of DNM with neutral or positive effects could facilitate future adaptation in populations, in humans, several negative effects of DNM have been identified. A number of DNM in humans were found to be associated with developmental disorders, including autism [3–5], schizophrenia [3], Miller syndrome and ciliary dyskinesia [6], and male infertility [7].

Breeding programmes, including those for livestock, rely on variation in the genome to select animals with desired characteristics, whether selection is for improving functional or production traits. However, the contribution of DNM to breeding goal traits has largely been ignored, especially in modern breeding programs that use genomic selection. Mulder et al*.* [8] showed that when animals had no own-performance record in simulated livestock breeding scheme’s, no selection pressure was put on DNM and therefore there was no opportunity to exploit mutational variance. Genomic selection with own-performance data in these simulations was able to best exploit mutational variance, even compared to traditional selection strategies based on mass selection or BLUP estimated breeding values, however, still lost total genic variance faster than the traditional selection strategies [8]. This loss in genetic variation in breeding programmes due to genomic selection may not be sustainable and should be of concern as it could have adverse consequences on the fitness of animals in breeding populations [8].

The number of direct mutation rate estimates by comparisons of genome sequences between subsequent generations in animals is increasing, but the estimates available are often based on a small sample size. Recently, a comparative analyses of germline mutation rate across vertebrates included estimates for single nucleotide DNM from 68 species [9]. Currently, for livestock, germline single nucleotide DNM rates per site per generation have been reported for chickens, pigs, goats, alpaca’s, and cattle, and range from 3.6 × 10^–9^ to 1.2 × 10^–8^ [9–11]. This corresponds to 4 to 35 single nucleotide DNM per generation per individual, which is much higher than the germline de novo structural variant (dnSV) rate of 0.108 per generation per individual reported in pigs [12]. Except for the rate of germline dnSVs estimated in the latter study, germline single nucleotide DNM rates reported in livestock were estimated based on only one to 10 trios and mutation rates tended to differ between animals. Therefore, to get accurate mutation rate estimates, studies with larger samples sizes are needed and of importance for the livestock industry, as DNM have the potential to contribute to total genetic variance in livestock breeding populations, given their short generation intervals and high selection intensities.

It is important to characterize DNM in livestock species to understand the genetic variation that is neglected by current selection strategies, to analyse when and where DNM occur, and to estimate their effects on fitness. Because unlike humans and wild animals, livestock species generally have short generation intervals, many progeny, and high quality phenotypic records, there is more power to detect fitness effects of DNM more quickly. DNM can have positive, negative, or neutral effects and it will be important to identify and maintain variants with positive or neutral (which could prove adaptive in the future) effects in populations, whereas variants with negative effects need to be detected and eliminated quickly.

Our objectives were to detect and validate single nucleotide DNM and estimate mutation rate in two commercial pig lines using high coverage whole genome sequence data from trios. We further aimed to characterise the validated DNM based on the specific base pair changes and their genomic context, and use predictive information to identify DNM that could have an effect on the fitness individuals.

## Materials and methods

### Samples and sequencing

A total of 46 pig trios were selected for sequencing, 22 trios from one commercial purebred pig line (15 sires, 22 dams, and 22 offspring of the trios, which will be referred to as probands from this point forward) and 24 trios from a second commercial purebred pig line (15 sires, 23 dams, and 24 probands). DNA was extracted from ear punches (all 59 line 1 samples, 36 line 2 samples), hair (11 line 2 samples), or semen (15 line 2 samples). Trios were selected for sequencing based on their availability of biological material (collected routinely for the breeding program), and each proband having > 30 offspring with phenotypic records and 50 K SNP genotype data. All samples were whole genome sequenced (mean sequencing depth = 32.4X) using Illumina HiSeq, with 150 bp read length and 300 bp fragment length. The paired-end reads were aligned to the pig reference genome (Sscrofa 11.1, GenBank assembly accession number GCF_000003025.6) with the Burrows-Wheeler Aligner (BWA-mem v.0.7.17) [13].

### DNM calling and filtering criteria

The variant calling pipeline was based on the “Germline short variant discovery best practices workflow” from GATK (https://gatk.broadinstitute.org/hc/en-us/articles/360035535932-Germline-short-variant-discovery-SNPs-Indels). Reads were aligned to the Sscrofa 11.1 assembly using BWA-mem (v.0.7.17) [13], duplicate reads were removed using samblaster [14], and the remaining reads were sorted and indexed with samtools [15]. Variants (single nucleotide variants (SNV) and indels) were called for each sample with GATK’s HaplotypeCaller, consolidated with GATK’s CombineGVCFs, and then joint-called with GATK’s GenotypeGVCFs [16, 17].

The de novo mutation (DNM) calling pipeline was based on the “Genotype refinement workflow for germline short variants” (https://gatk.broadinstitute.org/hc/en-us/articles/360035531432-Genotype-Refinement-workflow-for-germline-short-variants). The genotype posteriors and family priors were calculated using the called variants and pedigree information with GATK’s CalculateGenotypePosteriors [16]. Only SNV were kept and those with good quality were conserved (Phred-scaled probability that the call is incorrect (GQ) ≥ 20, quality normalized by the read depth (QD) < 4.0, phred-scaled probability there’s strand bias (FS) > 60.0, root mean squared mapping quality over all the reads at a site (MQ) < 40, test for comparing mapping qualities of reads supporting reference and alternative alleles (MQRankSum) < -2, test for comparing site positions of reference and alternative alleles in a read (ReadPosRankSum) < -8.0). Finally, all remaining SNV with allele count (AC) < max (4, 0.1% samples) were kept as high confidence DNM.

Further filtering criteria of DNM were implemented to ensure that candidate DNM were of high quality. The DNM needed to have a genotype quality (GQ) > 20, DP between 10 and 100 in the proband that they were found in, and > 20% of reads needed to support the DNM. For the sire and dam of the proband at the candidate DNM position, both parents needed a GQ > 20, a read depth between 10 and 100, and no reads supporting the DNM. Candidate DNM were only kept if they were detected in the proband but not in any other individuals in the population, except for half sibs or offspring of the proband that were also sequenced for this project. For each of the remaining candidates, as a final quality control step, raw reads (from BAM files) from the trio in which the mutation occurred were examined manually in JBrowse [18].

The transition transversion ratio for the candidate DNM was calculated by dividing the number of transition DNM by the number of transversion DNM. Transitions are point mutations where a purine has been interchanged with a purine, or a pyrimidine with a pyrimidine, while transversions are the change of a purine to a pyrimidine (or vice versa). Mutation rate per trio was calculated by dividing the number of DNM found in an individual by the proportion of the pig autosome that met the above filtering criteria. From the total set of 25,832,229 SNPs identified in the complete trio dataset, we filtered out SNPs that did not meet any of the above described criteria using the following command: ‘bcftools filter -e 'MIN(FORMAT/GQ) < 20 || MIN(FORMAT/DP) < 10 || MAX(FORMAT/DP) > 100 || QD <  = 4.0 || FS >  = 60.0 || MQ <  = 40 || MQRankSum <  = -2 || ReadPosRankSum <  = -8.0'’. The number of investigated SNPs was divided by the total number of SNPs to get the proportion of the genome that was investigated for de novo mutations. Next, we calculated the mutation rate by dividing the number of validated DNMs by the pig autosome size times the proportion of the genome considered.

For 19 trio’s 50K genotype data was available for the probands, allowing for testing of concordance between heterozygous genotype calls from the 50K SNPchip and the WGS set. Concordance rates were calculated as the proportion of heterozygous SNPs on the 50K SNPchip that were correctly called in the WGS dataset (see Additional file 1 Table S1). To get an estimate of the false negative rate, we examined WGS variants that overlapped with the 50K SNP set, focusing on Hom_RR, Het_RA, and Hom_AA genotype classes before and after applying the above filtering criteria. Results indicated that filtering disproportionately removed Hom_RR SNPs (about 5%) compared to Het_RA and Hom_AA SNPs (1–2%) in high-quality samples (see Additional file 1 Table S2). Overall, the filtering criteria did not show bias toward removing heterozygous SNPs and the false negative rate was relatively low in good quality samples.

### SNP genotyping

All the available parents, probands, and up to nine offspring of the proband were genotyped. Genotyping of predicted DNM was done using the PlexSeq™ genotyping platform (AgriplexGenomics.com). PlexSeq™ is an amplicon-based sequencing method for assaying up to 5000 SNPs. DNA of all individuals to be genotyped and sequence information (SNP alleles and 100 bp flanking the SNP) for the 704 single nucleotide DNM were submitted to Agriplex Genomics for primer design and genotyping. Genotyping was based on a two-step PCR followed by sequencing of the barcoded reads. PlexCall™ software was subsequently used to analyze allele frequencies, after which the SNP calls were compiled into a concise report. Genotyped DNM that were present in the proband, in at least one offspring of the proband, but not in the parents of the proband were considered as true germline DNM. Genotyped DNM that were present in the proband but not in the offspring (at least two genotyped offspring) and parents, were considered as possible somatic DNM.

### Validation of DNM based on whole genome sequence data

For probands with offspring sequenced (in this study or in previous studies), sequence data of the offspring were manually examined in JBrowse [18] to check for inherited DNM.

### Predicting effects of DNM

For each candidate DNM, the surrounding nucleotides, pig combined annotation dependent depletion (pCADD) scores [19] and the Ensembl Variant Effect Predictor (VEP) [20] results were compiled.

## Results

### Candidate DNM

A total of 704 single nucleotide DNM (see Additional file 1 Table S3) were detected in the probands. To validate the DNM identified using our DNM calling bioinformatics pipeline and to estimate a reliable germline DNM rate, we decided to genotype all 704 candidate DNM using the PlexSeq™ genotyping platform (Agriplex Genomics). For 675 DNM, primers could be designed and these were used to genotype the parents, the probands, and up to nine offspring of the proband in which the DNM were found (see Additional file 1 Table S4). Of the tested DNM, the assay failed for 73 DNM. Of the remaining 602 tested DNM, there was evidence that 404 (67.1%) were true DNM. Of these validated DNM, 395 were identified as true germline DNM, as they were validated in at least one offspring of the proband (see Additional file 1 Table S4). None of the validated DNM that were found in probands that had at least one sequenced half-sib available (see Additional file 1 Table S5) was detected in one of those half-sibs (see Additional file 1 Table S3), indicating that no germline mosaic DNM were identified.

**Fig. 1:**
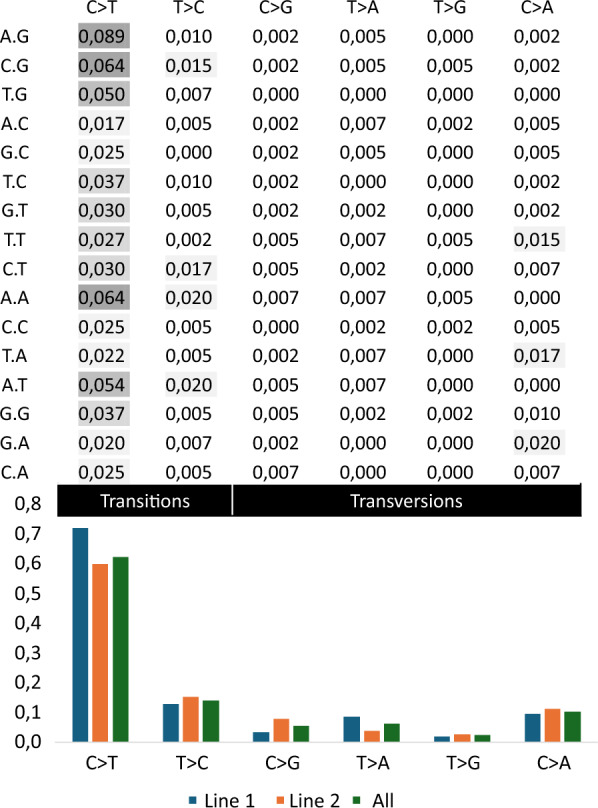
De novo mutational spectrum. The total proportion of validated de novo mutations (DNM) by base pair change (columns) and surrounding base pairs (rows), and the proportion of base pair changes by line (bar graph).

The total number of validated DNM in the two commercial pig lines, the average number of DNM per proband, and estimates of mutation rates and transition to transversion ratios (Ti/Tv) are presented in Table 1. More DNM were found in line 1 than line 2, although there were fewer trios in line 1. There were eight trios in line 2 for which we were unable to detect DNM due to sequence data quality, and these were excluded from further analyses. Overall, the profile of DNM found was similar for the two lines and reflected the pattern observed for the combined data (Fig. 1). Because there was no significant difference in mutation rates between the two lines (tested using two proportion z test, p < 0.01), nor in proportions of base pair changes (C > T: p = 0.337, T > C: p = 0.465, C > G: p = 0.043, T > A: p = 0.047, T > G: p = 0.617, C > A: p = 0.596), the data from the two lines were analyzed together.

**Table 1:** Summary of validated de novo mutations (DNM).

	Line 1	Line 2	Total
Number of DNM	213	191	404
Number of trios	18	20	38
Average number of DNM per proband	11.8	9.6	10.6
Mutation rate	6.4 × 10^–9^	6.3 × 10^–9^	6.3 × 10^–9^
Minimum mutation rate	0	3.3 × 10^–9^	0
Maximum mutation rate	1.1 × 10^–8^	1.2 × 10^–8^	1.2 × 10^–8^
Ti/Tv^1^	3.3	3.0	3.2

^1^Ti/Tv is the transition/transversion ratio. Transitions are point mutations where a purine has been interchanged with a purine, or a pyrimidine with a pyrimidine, while transversions are the change of a purine to a pyrimidine (or vice versa)

### Validation of DNM based on whole genome sequence data

The two pig lines included in our study are part of two separate commercial breeding programs. Therefore, we were able to use additional information that had been generated for these two lines as part of routine breeding practices or research and development programs. For example, line 2 also had key boars sequenced (short reads, 10 × coverage) outside of this study, allowing us to identify transmission of DNM to these key boars. Of the 191 DNM identified and validated in line 2, we found 42 in key boars that had been sequenced (see Additional file 1 Table S6). These 42 DNM were found in 1 to 20 sequenced animals. The 42 DNM found in sequenced key boars had a similar Ti/Tv ratio (2.8) as all 191 DNM from line 2 (3.0).

**Fig. 2:**
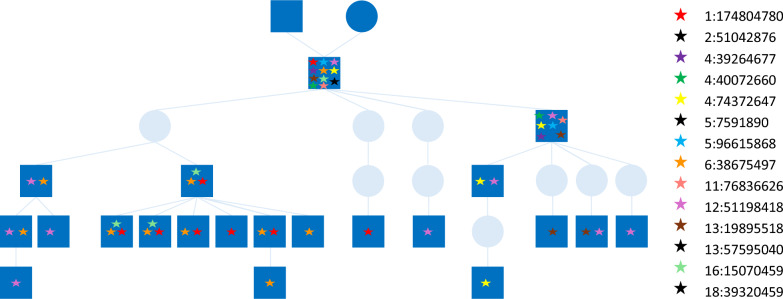
Germline mutations detected in the semen of a proband of line 2 and their inheritance in the following four generations. Sequenced animals (semen) in dark blue, squares represent males, circles represent females, and each de novo mutation is indicated with a star and position on the chromosome.

Figure 2 shows an example of one proband (generation one) for which we detected 14 DNM and the inheritance of 10 of these in the following four generations. In generation two, one animal had 7 DNM; in generation three, animals had 2 to 3 DNM; in generation four, animals had 1 to 3 DNM; and in generation five, individual animals had only 1 DNM remaining from the original 14 detected in generation one.

### Effects of DNM

**Fig. 3:**
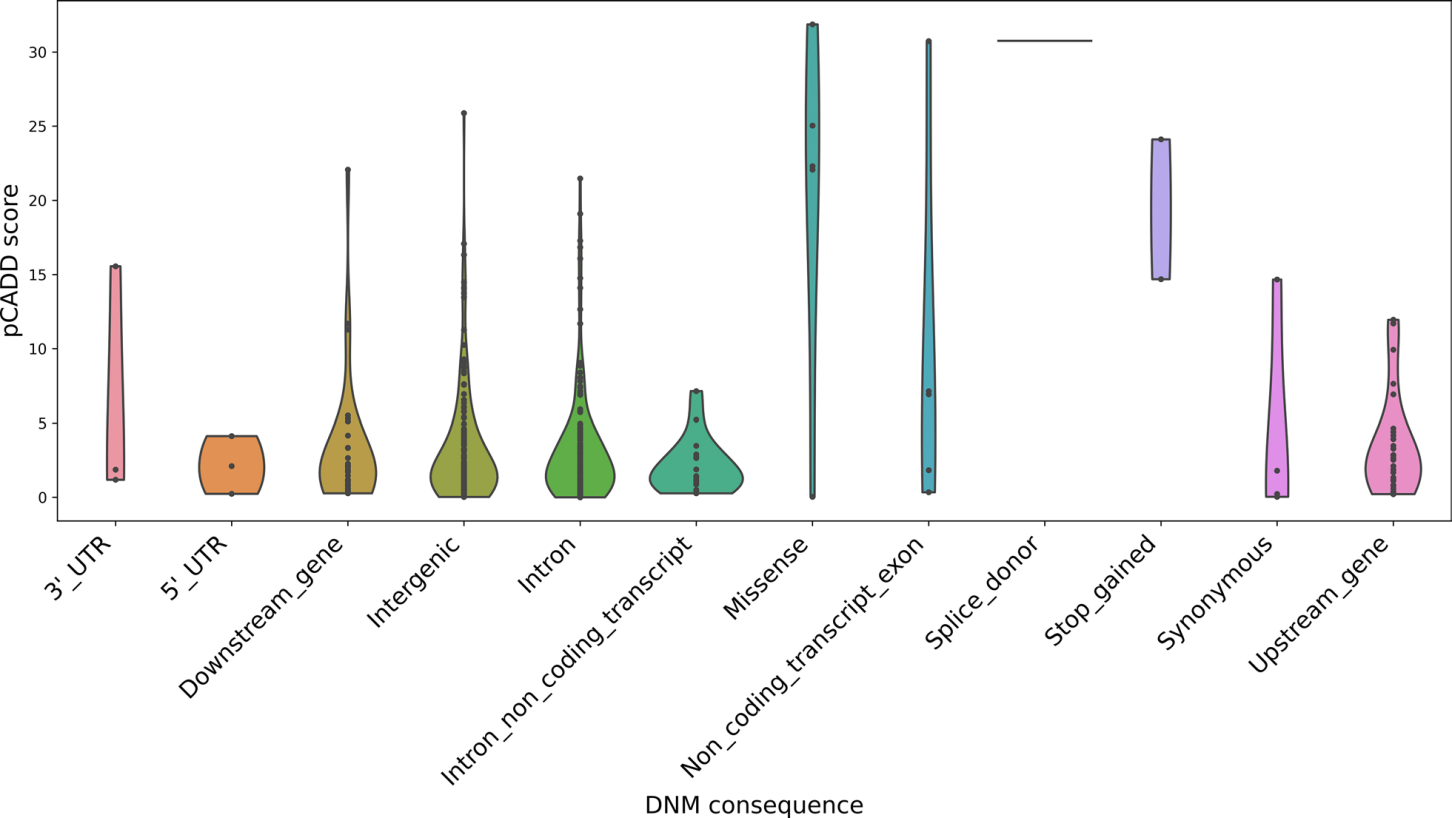
pCADD scores by consequence of all validated de novo mutations (DNM).

We used two tools to classify variants and predict their ability to disrupt a biological function of a genetic element: VEP annotation [20] and pCADD scores [19] (see Additional file 1 Table S3). DNM with higher pCADD scores can be prioritized as more likely having an effect on an individual, with pCADD scores of 10 and 20 corresponding to the 10 and 1% most deleterious substitutions, respectively [19]. By combining VEP and pCADD scores (Fig. 3), we identified several DNM that are interesting candidates for further study into their effects on complex traits, including missense, splice donor, stop gained, and upstream and downstream variants (see Additional file 1 Table S3).

The average pCADD score for the 42 DNM found in sequenced key boars was 2.29 and significantly differed (p = 0.03, tested using Welch’s t test) from the average pCADD score of 3.65 for all 191 validated DNM detected in line 2. Thirteen DNM were coding variants with an average pCADD score of 15, of which 8 were missense mutations with an average pCADD score of 20.6 (Fig. 3). Five of these missense mutations, located in the genes *SVEP1*, *SAMD9*, *MUSK* and *DOCK1*, were also predicted to be deleterious by SIFT (see Additional file 1 Table S3) and were predicted to impact all annotated transcripts of the reported genes and could therefore be potentially damaging.

## Discussion

In this study we identified and validated 404 DNM, of which nine might be somatic DNM, as they were validated in the proband but not in the 2 to 6 offspring of the proband that were genotyped. We presented a direct estimate of single nucleotide DNM rate in pigs and characterised the single nucleotide DNM detected. With the ever-increasing availability of sequence data, there are a growing number of direct estimates of mutation rate in species. In general, an average single nucleotide DNM rate of 6.3 × 10^–9^ per site per generation has been reported based on 36 mammalian species [9]. More specifically, in livestock, these estimates have ranged from 3.6 × 10^–9^ to 1.2 × 10^–8^ (chickens: 3.6 × 10^–9^; pigs: 3.6 × 10^–9^ and 4.3 × 10^–9^; goats: 5.3 × 10^–9^; alpaca’s: 9.4 × 10^–9^; and dairy cattle: 1.2 × 10^–8^) [9–11], but all were based on a small number of sequenced trios (one to nine). Our direct estimate of 6.3 × 10^–9^ for pigs fits into this range and our estimate based on a relative larger sample size adds more reliability on the direct estimates of the mutation rate in livestock, in particular in pigs. Moreover, because we use relatively stringent criteria to detect DNMs, our estimated mutation rate could still be underestimating the true DNM rate.

With the exception of humans, dairy cattle (M. Georges, personal communication), and now pig, all other direct mutation estimates have come from a very small number of sequenced trios (one to ten) [9, 21–25]. This presents a challenge, because the predicted mutation mechanisms are likely partly controlled by genetics and, therefore, variation in mutation rate between individuals and populations is expected. Mutations arise during the copying and recombination of DNA in the germline during meiosis and depends on the ability of a cell to make DNA repairs before these changes become permanent. Specifically, several biological mutation mechanisms have been postulated for humans: bulky DNA lesions that are resolved asymmetrically during transcription and replication, direction of the replication fork, replication timing, active demethylation in regulatory regions, and long interspersed nuclear elements, as well as a mechanism that is only attributed to oocytes [26]. Recombination rate has also been associated with mutation rate in humans [27] and recombination has been associated with several genes [28]. For the individual pig trios included in our study, we saw a range of mutation rates from 0 to 1.2 × 10^–8^, with a slightly higher mutation rate in one line (although based on our results this difference between lines is likely due to poor sequence quality in multiple samples in line 2, the line with fewer DNM). In humans variation in mutation rate has been observed between families [29]. However, mutation rate has also been shown to be relatively stable between human populations [30], with differences in mutation mechanisms observed instead [31]. In humans, the age of the father was shown to significantly increase the mutation rate, by 1.5 mutations per individual per year of age [32, 33]. Because variation in the age of the boars used in our study was small, we were not able to analyse whether age also affects mutation rates for males in pigs.

There is variation in mutational spectrum, the distribution of relative rates of different mutation types, across vertebrates and between human populations [9, 33], so we could expect there to be differences between the two pig lines in our study. Typically, in humans, the transition transversion (Ti/Tv) ratio of SNPs from sequencing data is 2 [34]. Although the number of possible transversions is twice as large as the number of possible transitions, transitions account for twice as many changes because of the increased amount of energy that is required to change ring structure. The Ti/Tv ratio in our study was 3.2 (3.3 in line 1 and 3.0 in line 2), which is higher than expected. Although a previous study [9] also found a higher Ti/Tv ratio in pigs (2.48) than in humans, our Ti/Tv ratio of 3.2 might indicate a bias, possibly because of the stringent filtering we applied. This would suggest that some Tv DNM were missed, and that the actual mutation rate is somewhat higher than the 6.3 × 10^–9^ based on the validated DNM.

**Fig. 4:**
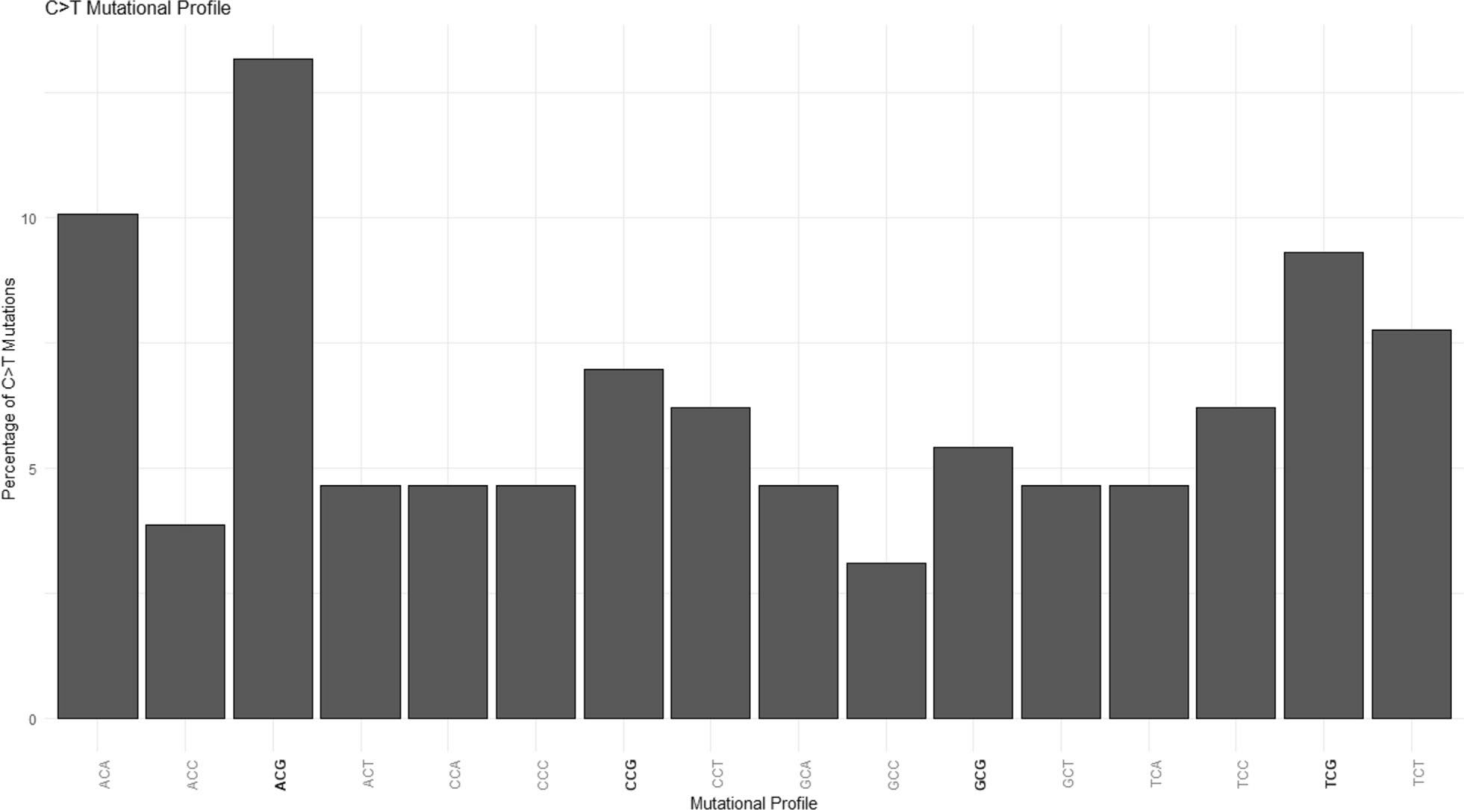
Mutational profile of validated C > T de novo mutations (DNM). Surrounding base pairs of the C > T mutations (x-axis) and the percentage of validated C > T mutations (y-axis) caused by spontaneous deamination of methylated CpG sites (ACG, CCG, GCG, TCG)

Within each of these types of point mutations, not every possible point mutation occurs equally. In a study of DNM in humans, there were 1.7 times more C > T than T > C transitions [35]. In contrast, in pigs, we found 4.4 times more C > T than T > C transitions. This is also reflected in the comparative study across vertebrates [9], which found 5.5 times more C > T than T > C transitions in pigs. The greater amount of C > T mutations in our study, could be partly explained by the spontaneous deamination of methylated CpG sites, as 35% of all C > T mutations occur at a CpG site (Fig. 4). The greater amount of C > T mutations in pigs compared to humans highlights that animal breeders cannot rely on mutation estimates in humans for predicting point mutations and for estimating mutation rate in other species, when considering the potential for including this type of information into breeding programs in the future.

All trios from line 1 and three trios from line 2 had only somatic tissue (ear punch for line 1 and ear punch or hair for line 2) available for sequencing. With our bioinformatics pipeline, we expect to be able to detect mosaic mutations that occurred early in the embryo of the probands. Hence, we also expect to pick up mosaic mutations in the proband’s somatic tissue. For true DNM that originated in one of the parents of the proband, we would expect a normal distribution with a mean read frequency that supports an alternate allele of 0.5. For the true validated DNM in our study, the average alternative read frequency was 0.475, which is significantly (one sample t-test, p < 0.01) different than the expected 0.5, most likely because of reference mapping bias [36, 37] or due to some DNM are actually mosaic mutations.

To study the effects of the DNM found in this study, we only sequenced pig trios for which the proband had at least 30 offspring that were genotyped with a SNP array. This implies that only probands with high estimated breeding values for traits under selection, were included here, as required for them to be eligible as breeding parents. However, this also means that our dataset suffers from pre-selection bias: none of the DNM we have detected is expected to have a large negative phenotypic effect in heterozygous state, because then the proband would not have had over 30 offspring genotyped. In humans, several DNM with severe consequences have been identified, including decreased male fertility and developmental disorders [3–7]. Such severe phenotypically visible consequences in commercial pigs would have prevented animals that carry such DNM from being mated or reproduce.

Regardless, we could have detected DNM that affect an individual in the homozygous state. Although we only considered DNM that were heterozygous in this study, it is possible to identify these potentially disruptive variants using tools such as pCADD scores, which predict the effect of base pair changes [19, 20]. Figure 3 identifies several DNM that were predicted to affect the fitness of an individual and this methodology could be used to prioritise the identified DNM for future studies.

Several studies suggest that a higher proportion of DNM originates from the paternal germline [10]. This may especially be relevant in livestock breeding programs, which often prioritize elite males. In such contexts, a greater paternal mutation rate can introduce a greater variety of new genetic variants into the population. However, this also carries a greater risk of incorporating deleterious variants from elite sires in the population.

Of the 191 DNM detected in line 2, we observed 42 in sequence data of related offspring, which provides additional independent validation for them being true DNM in the germline. In addition, it provides insight in what happens to DNM in a breeding program. In genomic selection, because most DNM have a neutral or very small effect and are not in linkage disequilibrium with SNPs on genotyping arrays, there is no selection pressure on the DNM. Indeed, in the example of Fig. 2, 11 of 14 DNM that were identified in the proband were lost after four generations. The contribution of mutations to genetic variance is believed to be the reason for the continued success of long-term genetic selection in plants and animals [38]. However, simulations have shown that genomic selection, when individuals do not have their own performance records, resulted in among the lowest mutational genic variance and total genic variance over 20 generations, when compared to other selection strategies [8]. Further research is needed into the long-term selection potential of genomic selection and strategies should be developed for maintaining total genetic variation (for example, Wray [38] proposed a method for BLUP to include mutation effects) and adopted in commercial breeding programs.

## Conclusions

In the current study, we detected and validated 404 single nucleotide DNM and estimated mutation rate using sequence data from 46 trios from two commercial pig lines. Our direct mutation rate estimate of 6.3 × 10^–9^ per site per gamete is in a similar range as the estimates reported in other livestock species, and with the relative larger sample size in this study, adds more reliability to the single nucleotide DNM rate in livestock. Using existing whole genome sequence data of offspring of some of the probands, we were able to follow the segregation of 42 DNM in up to four generations. The observed difference in mutational spectrum between pigs and humans suggests that animal breeders cannot rely on human mutation rate estimates for predicting point mutations and estimating mutation rate in other species, when considering the potential for including this type of information into breeding programs in the future. Finally, using predictive methods, we found several DNM that could have an impact on the fitness of an individual and should be prioritised when following up in studies estimating DNM effects. Overall, estimating mutation rate and characterising DNM in livestock species, as we have done with pigs in this study, provides basic knowledge needed to estimate DNM effects, and develop breeding methods that optimise the use of new genetic variation.

## Supplementary Information

Additional file 1: Table S1. Concordance of heterozygous genotype calls. Summarizes the concordance between genotype calls from the SNPchip and the WGS dataset per trio. Table S2. SNPs overlapping with 50K – pre and post filtering. SNPs in whole genome sequence data overlapping with the 50K SNP Chip before and after filtering criteria for the trios. Table S3. All 704 detected de novo mutations. All DNM detected including their physical position, line, read frequency, pCADD score, VEP, gene annotation, validation and the surrounding 200 base pairs. Table S4. Validation of the DNM. Validation of the DNM using the PlexSeq™ genotyping platform (Agriplex Genomics). Table S5. Summarizing table of DNM results per trio. Table summarizes the DNM results per trio, including the trio number, line number, number of sequenced offspring/half-sibs/full-sibs available, sample type, number of detected DNM, number of validated DNM, SNPs that meet filtering criteria, estimated DNM rate per trio. Table S6. The 42 DNM found in sequenced key boars. The 42 DNM found in sequenced key boars.

## Data Availability

The data that support the findings of this study are available from Hendrix Genetics B.V. and Topigs Norsvin but restrictions apply to the availability of these data, which were used under license for the current study, and so are not publicly available. Data are however available from the corresponding author upon reasonable request and with permission of Hendrix Genetics B.V. and Topigs Norsvin.
